# Supplementary material for: Diindolylmethane Derivatives: New Selective Blockers for T-Type Calcium Channels
Source: Membranes (Basel). 2022 Jul 30;12(8):749. doi: 10.3390/membranes12080749 (PMC9412534; doi:10.3390/membranes12080749)
Supplement: Supplementary file 1 [file membranes-12-00749-s001.zip › membranes-1845829-supplementary.pdf]

# SUPPORTING INFORMATION

## Diindolylmethane Derivatives: New Selective Blockers for T-type Calcium Channels

Dan Wang [1,2], Pratik Neupane [1], Lotten Ragnarsson [1], Robert J. Capon [1] and Richard J. Lewis [1,*]

1 Division of Chemistry and Structural Biology, Institute for Molecular Bioscience, the University of Queensland, Brisbane Qld 4072, Australia; danwang@ujs.edu.cn (D. W.); pratik.neupane.1984@gmail.com (P. N.); l.ragnarsson@imb.uq.edu.au (L. R.); r.capon@imb.uq.edu.au (R. J. C.); r.lewis@imb.uq.edu.au (R. J. L.)

2 Department of Chinese Medicine and Pharmacy, School of Pharmacy, Jiangsu University, Zhenjiang 212013, China; danwang@ujs.edu.cn (D. W.)

* Correspondence: r.lewis@imb.uq.edu.au; +617-3346-2984

### 1. General Experimental

All reagents were used as purchased from Sigma-Aldrich without further purification. Anhydrous solvents were obtained from PURE SOLV drying system and/or Sigma-Aldrich. Glass TLC Silica get 60 $F_{254}$ from Merk was used for thin layer chromatography. DAVISIL Chromatographic Silica (LC60A, 40-63 micron) was used for normal silica gel column chromatography. Flash column chromatography was performed on Biotage Isolera instrument using ethyl acetate (EtOAc) and petroleum ether as solvent. Compounds were subjected to Grace Pure SPE $C_{18}$ with mixtures of acetonitrile (MeCN) and water ($H_2O$) without trifloroacetic acid (TFA). SPE elutes were monitored using HPLC-MS (Agilent 1100 LC MSD model) with a standard gradient method. In addition, preparative HPLC was used for the purification of the compounds using water and acetonitrile containing 0.01% TFA as solvent. $^1$H, $^{13}$C, spectra were recorded on Bruker (600 MHz) spectrometers. $^1$H NMR was obtained in 600 Hz, and $^{13}$C NMR was obtained in 150 Hz. Data for $^1$H NMR spectra are reported as chemical shift (ppm), multiplicity (s = singlet, d = doublet; t = triplet; q = quartet; m = multiplet; dd = doublet doublet, br s = broad singlet) and coupling constant (*J* in Hz). High-resolution electrospray ionization mass spectrometry (ESIMS) spectra were obtained on a micrOTOF (Bruker) mass spectrometer by direct injection in MeCN at 3 μL/min using sodium formate clusters as an internal calibrant.

### 1.1 Synthesis of 3,3'-bisindolylmethanone (II)

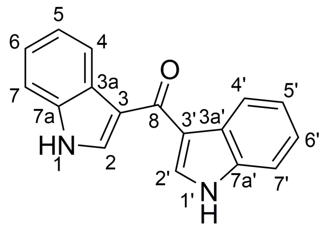


To a solution of indole-3-carboxylic acid (161 mg, 1 mmol) in anhydrous dichloromethane (20 mL) was added thionyl chloride (1.5 mmol) and catalytic amount of DMF and stirred at room temperature for 12 h. After the formation of indole 3 carbonyl chloride, solvent was evaporated and to the obtained residue was added 1.5 mL of anhydrous dichloroethane (DCE). To the above solution at 0°C under nitrogen was added a solution of indole (152 mg, 1.3 mmol) in anhydrous DCE. Zirconiumtetrachloride ($ZrCl_4$) (349.5 mg, 1.5 mmol) was added under a flow of nitrogen. The reaction temperature was then gradually increased to 30°C, and the reaction were continued at 30°C. After completion of the reaction as indicated by TLC (5 h), the resultant mixture was quenched with water (5 mL) and extracted with EtOAc (3 × 15 mL). The combined organic layer was washed with water (10 mL), dried with anhydrous

$Na_2SO_4$, and concentrated under vacuum. Recrystallization of the crude product with mass on methanol-ethyl acetate provided 3,3'-bisindolylmethanone **II** (156 mg 60% yield). $^1$H NMR (600 MHz, DMSO-$d_6$): δ 11.81 (br s, 2 H), 8.26 (d, $J$ = 7.8 Hz, 2 H), 8.16 (d, $J$ = 3 Hz, 2 H), 7.49 (d, $J$ = 7.8 Hz, 2 H), 7.22 (dd, $J$ = 7.8, 7.8, 2 H), 7.19 (dd, $J$ = 7.8, 7.8, 2 H). $^{13}$C NMR (150 MHz, DMSO-$d_6$): δ 184.5, 136.5, 131.9, 126.5, 122.4, 121.5, 120.9, 116.8, 111.8. HRMS (ESI) calcd for $C_{17}H_{13}N_2O$ $[M+H]^+$ 261.1022, found $m/z$ = 261.1025.

### 1.2 Synthesis of debromo-echinosulfone (III)

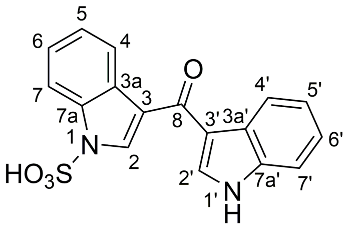


A mixture of **II** (10 mg, 0.04 mmol) and Py.$SO_3$ (12.7 mg, 0.08 mmol) in pyridine (0.1 mL) was heated at 120°C for 5 h. As no change was observed through TLC monitoring, additional 2 eq of Py.$SO_3$ was added and heated for 120°C for additional 6 h until the disappearance of the starting material 3,3'-diindolylmethanone. After being cooled to room temperature, the mixture was diluted with water (2 mL), was neutralized with 1 M HCl, and was concentrated under reduced pressure. The residue was subjected to reverse phase column chromatography and eluted with mixture of water and acetonitrile. Concentration of the eluate afforded debromo-echinosulfone **III** (5.2 mg, 38% yield) as an orange solid. $^1$H NMR (600 MHz, DMSO-$d_6$): δ 11.82 (br s, 1 H), 8.22 (d, $J$ = 8.4 Hz, 1 H), 8.20 (d, $J$ = 7.8 Hz, 1 H), 8.06 (br s, 2 H), 7.88 (d, $J$ = 8.4 Hz, 1 H), 7.51 (d, $J$ = 7.8 Hz, 1 H), 7.26 (dd, $J$ = 8.4, 7.8 Hz, 1 H), 7.22 (m, 2 H), 7.18 (dd, $J$ = 8.4, 7.8 Hz, 1 H).$^{13}$C NMR (150 MHz, DMSO-$d_6$): δ 184.5, 136.6, 135.1, 132.3, 132, 127.3, 126.4, 123, 122.7, 121.7, 121.4, 121.3, 121.2, 116.7, 115.7, 113.9, 112.2. HRMS (ESI) calcd for $C_{17}H_{10}N_2O_4S$ $[M+H]^+$ 341.0557, found $m/z$ = 341.0554.

### 1.3 Synthesis of 6,6'-dibromo 3,3'-bisindolylmethanone (IV)

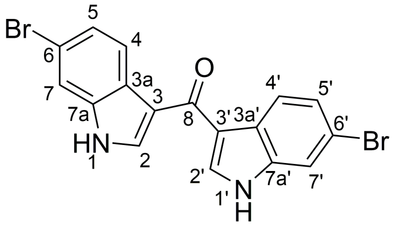


A solution of 6-bromoindole-3-carboxylic acid (98 mg, 0.5 mmol), thionyl chloride (1.5 mmol) and catalytic amount of DMF in anhydrous DCM (20 mL) was stirred at room temperature for 12 h. The solvent was removed *in vacuo* and 15 mL of anhydrous DCE was added to residue. To this solution, 6-bromo indole (117 mg, 0.6 mmol) and $ZrCl_4$ (162 mg, 0.7 mmol) was added at 0°C under nitrogen. The reaction temperature was then gradually increased to 30°C, and reaction stirred for 5 h. The resultant mixture was quenched with water (5 mL) and extracted with EtOAc (3 × 15 mL). The combined organic layer was washed with water (10 mL), dried with anhydrous $Na_2SO_4$, and solvent removed *in vacuo*. Recrystallization of residue in MeOH and EtOAc afforded 6,6'-dibromo 3,3'-bisindolylmethanone **(IV)** (124 mg 60% yield). $^1$H NMR (600 MHz, DMSO-$d_6$): δ 11.98 (br s, 2 H), 8.23 (d, $J$ = 3 Hz, 2 H), 8.18 (d, $J$ = 8.4 Hz, 2 H), 7.68 (d, $J$ = 1.8 Hz, 2 H), 7.32 (dd, $J$ = 8.4, 1.8, 2 H). $^{13}$C NMR (150 MHz, DMSO-$d_6$): δ 183.9, 137.4, 132.9, 125.5, 124, 123.1, 116.5, 115.2, 114.5. HRMS (ESI) calcd for $C_{17}H_{10}N_2O_4S$ $[M+H]^+$ 415.9160, found $m/z$ = 415.9160.

### 1.4 Synthesi of 6,6'-dibromo 3,3'-bisindolylmethanone-1*H*-indole-1-sulfonic acid (2.14b) (V)

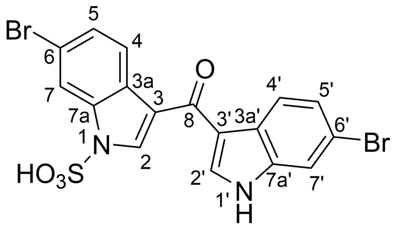

A mixture of **IV** (10 mg, 0.024 mmol) and Py.$SO_3$ (8 mg, 0.05 mmol) in pyridine (0.1 mL) was heated for 120°C for 8 h. After being cooled to room temperature, the mixture was diluted with water (2 mL), was neutralized with 1M HCl, and was concentrated under reduced pressure. The purification of dried reaction mixture was subjected to SPE reverse phase column chromatography and eluted with mixture of water and acetonitrile to afford **V** (2.5 mg, 20% yield) as a brown solid. $^1$H NMR (600 MHz, DMSO-$d_6$): δ 11.96 (br s, 1 H), 8.16 (d, $J$ = 2.7 Hz, 1 H), 8.15 (d, $J$ = 8.4 Hz, 1 H), 8.14 (d, $J$ = 8.1 Hz, 1 H), 8.08 (s, 1 H), 8.03 (br s, 1 H), 7.70 (br s, 1 H), 7.37 (d, $J$ = 8.6 Hz, 1 H), 7.32 (d, $J$ = 8.4 Hz, 1 H). $^{13}$C NMR (150 MHz, DMSO-$d_6$): δ 183.7, 137.5, 135.6, 133, 132.7, 126.2, 125.3, 124.6, 124.1, 123, 116.4, 116.3, 115.7, 115.4, 115.2, 114.7. HRMS (ESI) calcd for $C_{17}H_{10}N_2O_4S$ $[M+H]^+$ 496.8728, found *m/z* = 496.8728.

### 1.5 Synthesis of ethyl 2-(1*H*-indol-3-yl)-2-oxoacetate (VI)

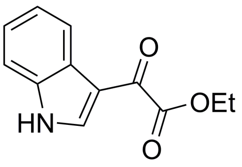

To a stirred solution of indole-3-carboxylic acid (1.01 g, 8.66 mmol) in anhydrous $Et_2O$ (17 mL), pyridine (0.95 mL, 11.7 mmol) was added and stirred for 5 min. A solution of ethyl chlorooxoacetate (1.2 mL, 10.7 mmol) in anhydrous $Et_2O$ (3 mL) at 0°C was added over a period of 15 min. The reaction mixture was stirred at 0°C for 2 h and filtered. The resulting solid was then washed with cold $Et_2O$ and water, dried over high vacuum to give **VI** (1.52 g, 81% yield) as a light brown powder. $^1$H NMR (600 MHz, DMSO-$d_6$): δ 12.39 (br, 1H,), 8.42 (d, $J$ = 3.3 Hz, 1 H), 8.18−8.13 (m, 1 H), 7.57−7.53 (m, 1 H), 7.32−7.24 (m, 2 H), 4.36 (q, $J$ = 7.1 Hz, 2 H), 1.34 (t, $J$ = 7.1 Hz, 3 H). $^{13}$C NMR (150 MHz, DMSO-$d_6$): δ 179.1, 163.6, 138.3, 136.7, 125.5, 123.9, 122.9,121.1, 112.8, 112.4, 61.6, 14.0. MS (ESI-TOF, m/z): calculated for $C_{12}H_{11}NO_3$ $[M + H]^+$ 218.0812; found 218.0796.

### 1.6 Synthesis of ethyl 2-oxo-2-(1-tosyl-1*H*-indol-3-yl)acetate (VII)

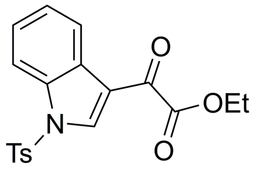

To a stirred solution of **VI**(65 mg, 0.3 mmol) in $CH_2Cl_2$ (15 mL), triethylamine (0.8 mmol, 80 mg) and DMAP (12 mg, 0.1 mmol,) was added. To this mixture, p-toluenesulfonyl chloride (0.8 mmol, 152 mg) was added and stirred at room temperature for 6 h till the disappearance of the starting material. The solvent was removed under reduced pressure and residue was purified by flash chromatography (EtOAc/ ether 1:4) to give **VII** (76 mg, 69% yield). $^1$H NMR (600 MHz, $CDCl_3$): δ 8.85 (s, 1 H); 8.36 (m, 1 H), 7.97 (m, 1 H), 7.88 (d, $J$ = 8.4 Hz, 2 H), 7.38 (m, 2 H), 7.27 (m, 2 H,), 4.4 (q, $J$ = 7.2 Hz, 2 H), 2.37 (s, 3 H), 1.45 (t, $J$ = 7.0 Hz, 3 H). $^{13}$C NMR (150 MHz, $CDCl_3$): δ 178.6, 161.5, 146.1, 136.7, 134.3, 134.1, 130.2, 127.5, 127.2, 126.0, 125, 122.7, 117, 113.0, 62.4, 29.7, 21.6, 14.0. HRMS (ESI) calculated for $C_{19}H_{18}N_2O$ $[M+H]^+$ 372.0906, found *m/z* = 372.0906.

### 1.7 Synthesis of 1,2-di(1*H*-indol-3-yl)ethane-1,2-dione (VIII)

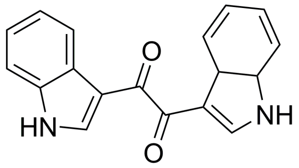

To a stirred solution of indole (234 mg, 2 mmol,) in dry $Et_2O$ (10 mL) at 0°C under an atmosphere of argon gas, oxalyl chloride (378 mg, 3 mmol) was added dropwise. The mixture was stirred for 2 h, resulting in a bright yellow precipitate. The solvent was removed by evaporation and dichloroethane (10 mL) was added. At 0°C indole (234 mg, 2 mmol) and $AlCl_3$ (399 mg, 3 mmol) were added and temperature gradually increased to room temperature. After the completion of reaction (2 h), the reaction was quenched with water (10 mL) and the organic fraction was extracted with EtOAc (20 × 3 mL). The organic phase was dried on $MgSO_4$ and filtered. The solvent was removed under vacuum, yielding **VIII** as a white reddish brown solid (403 mg, 70% yield). $^1$H NMR (DMSO-*d6*, 600 MHz): δ 12.23 (s, 2 H), 8.77- 8.26 (m, 2 H), 8.22 (s, 1 H), 8.21 (s, 1 H), 7.54 -7.53 (m, 2 H), 7.30 -7.27 (m, 4 H). $^{13}$C NMR (150 MHz, DMSO-*d6*): δ 188.7, 137.3, 136.7, 125.5, 123.4, 122.4, 121.2, 112.5, 112.4; HRMS (ESI) calcd for $C_{18}H_{13}N_2O_2$ $[M+H]^+$ 289.0899, found m/z = 289.0897.

### 1.8 Synthesis of bis(1-benzyl-1*H*-indol-3-yl)methanone (IX)

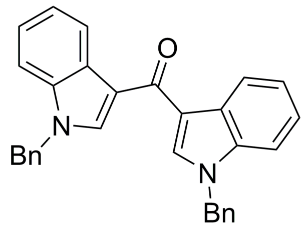

To a solution of **II** (78 mg, 0.3 mmol) in anhydrous THF (10 mL) was added KOH (1.2 mmol) and 4 mL of DMSO and stirred at room temperature. Benzyl bromides (1.2 mmol) was added to reaction mixture and stirred at room temperature for 6 h till the disappearance of the starting material. Upon finished the reaction mixture was quenched with water (10ml) and extracted by EtOAc (3 × 15 mL). The combined organic layer was dried over $Na_2SO_4$, filtered, concentrated in *vacuo* and purified by flash chromatography (30% EtOAc/ether) afforded benzyl-protected bisindole **IX** (66mg, 50% yield) $^1$H NMR (600 MHz, $CDCl_3$): δ = 8.49 (m, 2 H), 8.35 (m, 2 H); 7.55 (m, 2 H), 7.30 (m, 14 H), 5.60 (m, 4 H). $^{13}$C NMR (150 MHz, DMSO-*d6*): δ 137.5, 137.0, 127.6, 127.3, 126.40, 125.62, 124.4, 120.5, 118.4, 117.9, 113.8, 108.5, 48.84. HRMS (ESI) calculated for $C_{31}H_{25}N_2O$ $[M+H]^+$ 441.1967, found *m/z* = 441.1967.

### 1.9 Synthesis of bis(1-tosyl-1*H*-indol-3-yl)methanone (X)

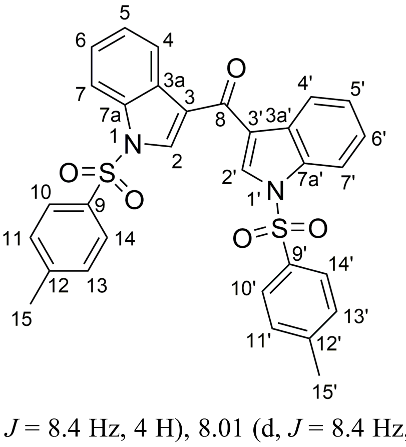

To a stirred solution of **II** (78 mg, 0.3 mmol) in $CH_2Cl_2$ (3 mL), triethylamine (0.8 mmol, 80 mg) and DMAP (12 mg, 0.1 mmol,) was added. To this mixture p-toluenesulfonyl chloride (0.8 mmol, 152 mg) was added and stirred at room temperature for 6 h till the disappearance of the starting material. The solvent was removed under reduced pressure and residue was purified by flash chromatography (EtOAc/ ether 1:4) to give **X** (170 mg, 100% yield). $^1$H NMR (DMSO-*d6*, 600 MHz): δ 8.66 (s, 2 H), 8.12 (d, *J* = 7.8 Hz, 2 H), 8.03 (d, *J* = 8.4 Hz, 4 H), 8.01 (d, *J* = 8.4 Hz, 2 H), 7.47 (dd, *J* = 8.4, 7.2 Hz, 2 H), 7.42 (dd, *J* = 8.4, 4 H), 7.41 (dd, *J* = 7.8, 7.2 Hz, 2 H), 2.32 (s, 6 H). $^{13}$C NMR (150 MHz, DMSO-*d6*): δ 183.7, 146.3, 134.2,

133.5, 130.4, 127.8, 127.3, 125.9, 124.7, 122, 120.8, 113.1, 21.1. HRMS (ESI) calcd for $C_{31}H_{24}N_2NaO_5S_2$ $[M+Na]^+$ 519.1019, found *m/z* = 519.1016.

### 1.10 Synthesis of 3,3'-carbonylbis(1*H*-indole-1-sulfonic acid) (XI)

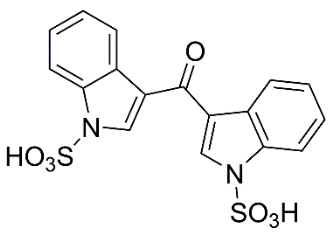

A mixture of **II** (10 mg, 0.04 mmol) and Py.$SO_3$ (50 mg, 0.32 mmol) in pyridine (0.1 mL) was heated at 120°C for 20 h. After being cooled to room temperature, the mixture was diluted with water (2 mL), was neutralized with 1 M HCl, and was concentrated under reduced pressure. The residue was purified by reverse phase SPE using mixture of water and acetonitrile afforded the sulfonated compound **XI** (4 mg, 21% yield). $^1H$ NMR (600 MHz, DMSO): δ 8.06 (br s, 2 H), 8.20 (d, *J* = 8.20 Hz, 2 H), 7.88 (d, *J* = 8.4 Hz, 2 H), 7.26 (t, *J* = 7.8 Hz, 2 H), 7.22 (m, 2 H). $^{13}C$ NMR (150 MHz, DMSO): δ 184.5, 135.1, 132.3, 127.3, 123.0, 121.7, 121.3, 115.7, 113.9. HRMS (ESI) calculated for $C_{17}H_{10}N_2O$ $[M+H]^+$ 420.0086, found *m/z* = 420.0086.

## 2. Cell Viability MTT Assay

### 2.1 Effects of the synthetic compounds on cancer cell viability in MTT assay

Compounds (50 µM) with significant anti-proliferative activities are highlighted in Table S1. Based on the promising data obtained from the one-concentration antiproliferative effects, we decided to examine the concentration response effects of DIM, DIM-one, IV and V. The concentration dependent effects of the four compounds on the four cancer cell lines after 72 h treatment are concluded in Table S2.

**Table S1.** Effects of the synthetic compounds on cancer cell viability in MTT assay

| Compounds | MCF-7 | T-47D | A549 | HT-29 |
|---|---|---|---|---|
| | Cell viability (%) (*n* = 4) | | | |
| DIM | 3.65 ± 0.24 | 18.87 ± 1.15 | 55.65 ± 1.13 | 25.29 ± 2.44 |
| DIM-one | 52.26 ± 3.12 | 50.64 ± 2.08 | 81.26 ± 3.00 | 5.95 ± 0.19 |
| III | 84.87 ± 5.75 | 79.71 ± 3.74 | 210.91 ± 4.69 | 21.62 ± 1.10 |
| IV | 6.35 ± 0.46 | 6.14 ± 0.55 | 2.92 ± 0.29 | 0.85 ± 0.21 |
| V | 29.52 ± 0.19 | 31.37 ± 0.43 | 14.14 ± 0.15 | 0.93 ± 0.06 |
| VIII | 43.29 ± 1.25 | 64.17 ± 1.67 | 52.59 ± 1.70 | 57.31 ± 5.01 |

**Table S2.** Concentration dependent effects of DIM, DIM-one, IV and V on cancer cell viability after 72 h treatment measured in the MTT assay

| 72 h Treatment | MCF-7 | T-47D | A549 | HT-29 |
|---|---|---|---|---|
| | $IC_{50}$ (μM) ($n$ = 3) | | | |
| DIM | 27 | 36 | 47 | 33 |
| DIM-one | 42 | 44 | 66 | 20 |
| IV | 6 | 4 | 3 | 1 |
| V | 51 | 38 | 27 | 15 |

Treatment of the four cell lines with IV for 24 h, 48 h, and 72 h were further investigated (Figure S1), and $IC_{50}$ values concluded in Table S3, one decimal place kept for a better comparison. Instead of a continually increasing effectiveness with elongated treatment times, 48 h and 72 h treatment of IV generally showed similar antiproliferative effects on all four cell lines.

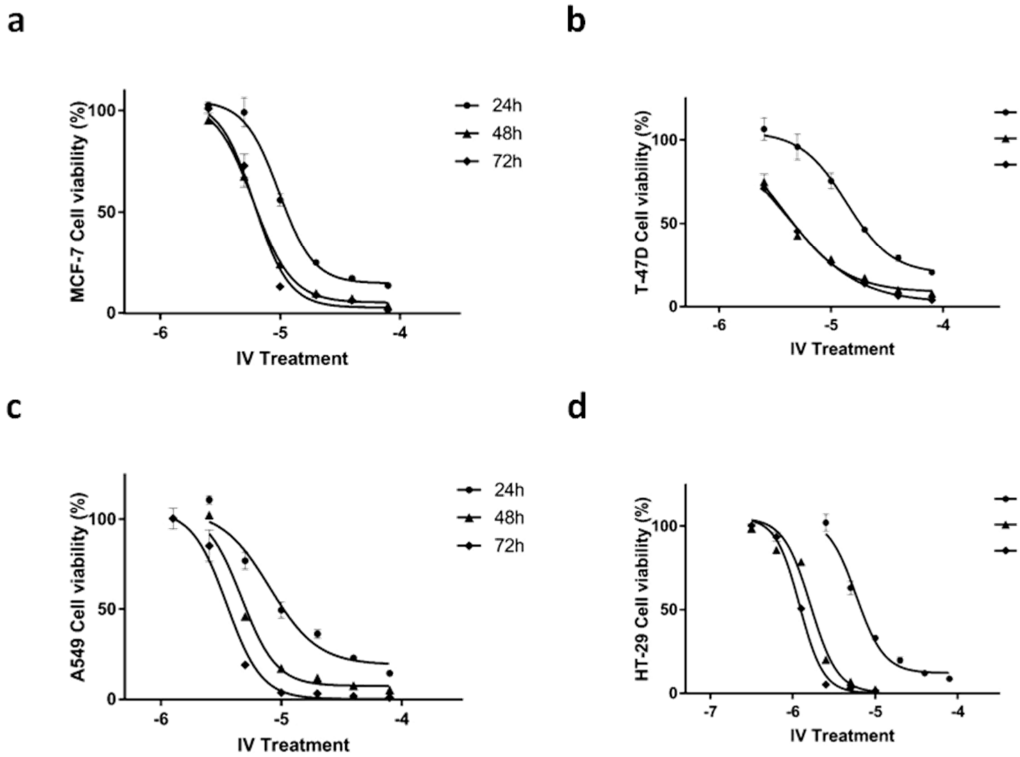


**Figure S1. Cytotoxic effects of IV on the MCF-7, T-47D, A549 and HT-29 cell lines after 24 h, 48 h, and 72 h treatment measured in the MTT assay.**

(**a**) Concentration dependent effects of IV treatment on MCF-7 cell viability. (**b**) Concentration dependent effects of IV treatment on T-47D cell viability. (**c**) Concentration dependent effects of IV treatment on A549 cell viability. (**d**) Concentration dependent effects of IV treatment on HT-29 cell viability. Error bars represent the SEM of one separate experiment performed in triplicate.

**Table S3.** Concentration dependent effects of IV on cancer cell viability after 24 h, 48 h, and 72 h treatment measured in MTT assay

| Treatment | | MCF-7 | T-47D | A549 | HT-29 |
|---|---|---|---|---|---|
| | | $IC_{50}$ (μM) ($n$ = 3) | | | |
| IV | **24 h** | 9.8 | 13.8 | 8.0 | 5.9 |
| | **48 h** | 6.0 | 3.9 | 4.7 | 1.6 |
| | **72 h** | 6.1 | 4.1 | 3.5 | 1.2 |
